# Prenatal lead, cadmium and mercury exposure and associations with motor skills at age 7 years in a UK observational birth cohort

**DOI:** 10.1016/j.envint.2018.04.032

**Published:** 2018-08

**Authors:** Caroline M. Taylor, Alan M. Emond, Raghu Lingam, Jean Golding

**Affiliations:** a Centre for Child and Adolescent Health, Bristol Medical School, University of Bristol, UK; b School of Women's and Children's Health, UNSW, Sydney, Australia

**Keywords:** ALSPAC, Avon Longitudinal Study of Parents and Children, Movement ABC, Movement Assessment Battery for Children, ALSPAC, Lead, Mercury, Cadmium, Motor skills, Child, Pregnancy

## Abstract

**Background:**

Lead and mercury are freely transferred across the placenta, while cadmium tends to accumulate in the placenta. Each contributes to adverse neurological outcomes for the child. Although prenatal heavy metal exposure has been linked with an array of neurodevelopmental outcomes in childhood, its association with the development of motor skills in children has not been robustly studied.

**Aims/objective:**

The aim of the present study was to investigate the association between prenatal exposure to lead, cadmium and mercury, measured as maternal blood concentrations during pregnancy, and motor skills, measured as subtests of the Movement Assessment Battery for Children (Movement ABC) at age 7 years in a large sample of mother–child pairs enrolled in a UK observational birth cohort study (Avon Longitudinal Study of Parents and Children, ALSPAC).

**Methods:**

Whole blood samples from pregnant women enrolled in ALSPAC were analysed for lead, cadmium and mercury. In a complete case analysis (n = 1558), associations between prenatal blood concentrations and child motor skills assessed by Movement ABC subtests of manual dexterity, ball skills and balance at 7 years were examined in adjusted regression models. Associations with probable developmental coordination disorder (DCD) were also investigated.

**Results:**

The mean prenatal blood levels were: lead 3.66 ± 1.55 μg/dl; cadmium 0.45 ± 0.54 μg/l; mercury 2.23 ± 1.14 μg/l. There was no evidence for any adverse associations of prenatal lead, cadmium or mercury exposure with motor skills measured at age 7 years with Movement ABC subtests in adjusted regression models. Further, there were no associations with probable DCD.

**Conclusions:**

There was no evidence to support a role of prenatal exposure to heavy metals at these levels on motor skills in the child at age 7 years measured using the Movement ABC. Early identification of symptoms of motor skills impairment is important, however, to enable investigation, assessment and treatment.

## Highlights

• The association of in utero heavy metal exposure with motor skills in children is unclear.
• Whole blood samples from pregnant women were analysed for lead, cadmium and mercury.
• Motor skills were assessed by manual dexterity, ball skills and balance ability at 7 years.
• There was no evidence for a role of prenatal exposure on motor skills in the child.
• Early identification of symptoms of motor skills impairment is still important.

## 1 Introduction

Lead, cadmium and mercury are toxic metals that are widespread in the environment from natural and anthropogenic sources. Lead is transferred freely across the placenta (the ratio of fetal:maternal blood lead is about 0.7–0.9 (Rudge et al., 2009; Schell et al., 2003)) and across the blood–brain barrier, as is mercury; transfer of cadmium is less marked but it tends to accumulate in the placental tissue where it may interfere with zinc transport and affects endocrine hormone synthesis and cellular functions (Caserta et al., 2013). In utero exposure to these toxic metals may therefore contribute to adverse neurodevelopmental outcomes: the fetus is particularly vulnerable to the effects of toxic metals because of high rates of cell division and differentiation. Thus, relatively low levels of exposure that do not greatly harm the mother may have an effect on the development of the fetus, and on subsequent development and behaviour during childhood. Neurodevelopmental outcomes include cognitive, sensory and motor functions: adverse effects of postnatal exposures to lead, cadmium and mercury on cognition (Counter et al., 2006; Crump et al., 2013; Sanders et al., 2015) and on sensory functions such as hearing (Choi et al., 2012; Shargorodsky et al., 2011), have been relatively well studied. Motor skills have been less well studied, and with conflicting results (Dietrich et al., 1993; Ohlander et al., 2016; Wasserman et al., 2000). With regard to prenatal exposures, there are few studies of the effects on motor skills, particularly for lead and cadmium. The results are conflicting, with some studies finding adverse associations (Baghurst et al., 1995; Bonithon-Kopp et al., 1986; Debes et al., 2006; Jedrychowski et al., 2006; Kim et al., 2013), some finding no associations (Bellinger et al., 1984; Rothenberg et al., 2016; Snoj Tratnik et al., 2017; van Wijngaarden et al., 2013), with others finding a mixed picture possibly dependent on the timings of measurements of the exposure and of the outcome (Cordier et al., 2002; Davidson et al., 2000; Jedrychowski et al., 2007; Prpic et al., 2017; Shah-Kulkarni et al., 2016).

As a result of progress in abatement measures in high-income countries, the main sources of exposure to lead in developed countries are water, dust and soil, food and drink (European Food Safety Authority Panel on Contaminants in the Food Chain, 2010), and cigarette smoke (Taylor et al., 2013). This has been achieved primarily through the removal of lead from petrol and paint, although paint on some playground equipment and street furniture in the UK still contains relatively high levels of lead (Turner et al., 2016; Turner and Solman, 2016). Cadmium exposure is generally associated with battery manufacture and recycling, but exposure also arises through fossil fuel combustion, waste incineration, and manufacturing processes such as those for cement, iron and steel. Smoking tobacco, however, is the most important source of cadmium in the general population (Bernhard et al., 2005). For non-smokers, the primary source of exposure is through diet, with grains and grain products, vegetables and vegetable products, and starch roots and tubers making the greatest contributions to total ingestion (European Food Safety Authority Panel, 2012). Mercury is present in the environment through a variety of sources: mercury-containing aerosols are released from volcanic activity and from the weathering of rocks, while human activities such as mining and manufacturing processes also contribute to levels in the environment (Hylander and Meili, 2003; Nriagu and Becker, 2003). Further exposure to mercury at a population level occurs through diet, particularly from fish that are long-lived and high in the food chain (Castano et al., 2015; Golding et al., 2013), and dental amalgam (Golding et al., 2016).

The aim of the present study was to investigate the association between prenatal exposure to lead, cadmium and mercury, measured as maternal blood concentrations during pregnancy, and motor skills, measured as subtests of the Movement Assessment Battery for Children (Movement ABC) at age 7 years in a large sample of mother–child pairs enrolled in a UK observational birth cohort study (Avon Longitudinal Study of Parents and Children, ALSPAC). A secondary aim was to test associations with probable developmental coordination disorder (DCD).

## 2 Methods

**Fig. 1:**
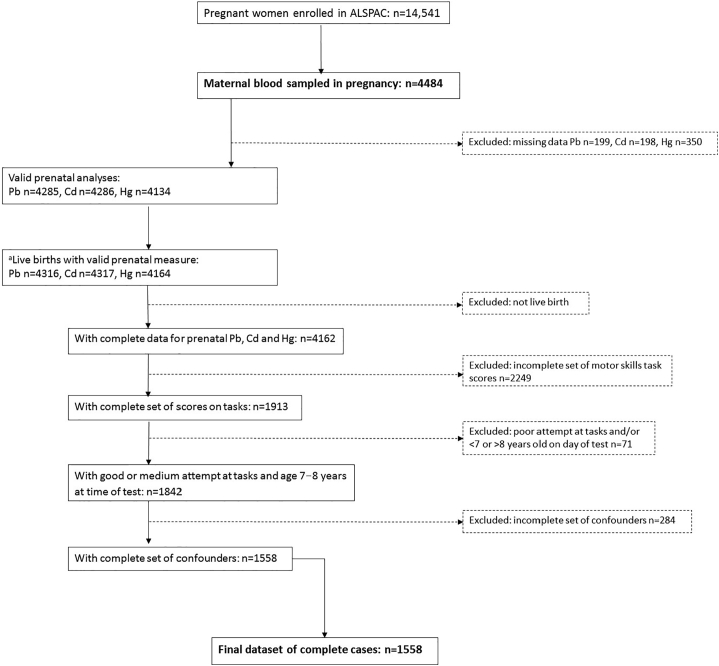
Study flowchart. ^a^Number of live births with valid prenatal measures is greater than number of valid prenatal analyses because of multiple births.

The sample for this analysis was derived from ALSPAC, which is a UK-based birth cohort set up to investigate environmental and genetic influences on health and disease. ALSPAC recruited 14,541 pregnant women resident in Avon, UK with expected dates of delivery between 1st April 1991 and 31st December 1992. The cohort profile is described in full detail elsewhere (Boyd et al., 2013; Fraser et al., 2013). The study website contains details of all the data that are available through a fully searchable data dictionary, which is accessible at http://www.bristol.ac.uk/alspac/researchers/access/. Ethics approval for the study was obtained from the ALSPAC Ethics and Law Committee and the Local Research Ethics Committees. The study flow chart for complete cases is shown in Fig. 1.

### 2.1 Exposure: collection, storage and analysis of blood samples

Whole blood samples were collected in acid-washed vacutainers (Becton and Dickinson, Oxford, UK) by midwives as early as possible in pregnancy. The median gestational age at the time of blood sampling was 11 weeks (interquartile range 9–13 weeks). Whole blood samples were stored in the original tube at 4 °C at the collection site before being transferred to the central Bristol laboratory within 1–4 days. Samples were at ambient temperature during transfer (up to 3 h). They were then stored at 4 °C until analysis.

Inductively-coupled plasma mass spectrometry in standard mode (R. Jones, Centers for Disease Control and Prevention (CDC), Bethesda, MD, USA; CDC Method 3009.1) was used to measure blood levels with appropriate quality controls (Iles-Caven et al., 2016; Taylor et al., 2013; Taylor et al., 2017). The analyses were completed on samples from 4285 women for lead, 4286 women for cadmium and 4134 women for mercury. One sample had a lead level below the limit of detection (0.24 μg/dl), 1119 for cadmium (0.20 μg/l) and three for mercury (0.24 μg/l). These samples were assigned a value of 0.7 times the lower limit of detection (limit of detection/√2 to reflect the log-normal distribution (Centers for Disease Control and Prevention, 2005; Hornung and Reed, 1990)).

Prenatal lead concentrations were categorised as <5 or ≥5 μg/dl based on the US “reference value” (Centers for Disease Control and Prevention, 2012). As there are no widely accepted reference values for cadmium or mercury, these were categorised according to the median values for the dataset with either all available cases or complete cases.

### 2.2 Outcomes

#### 2.2.1 Movement ABC subtests

The ALSPAC Coordination Test was derived from subtests of the Movement ABC (Henderson and Sugden, 1992), carried out in research clinics when the child was about 7.5 years old. It was conducted in rooms adapted for the study by trained examiners from ALSPAC. Parents accompanied the children but were not allowed to help them. In each case the tester also rated the child's attempt at the task as good, medium or poor. Details of the methodology for each subtest (balance: heel to toes steps subtest; ball skills: beanbag subtest; manual dexterity: placing pegs subtest; manual dexterity: threading lace subtest) are described in detail in Taylor et al. (2018). Subtest results were categorised as follows: heel to toe subtest 15 steps completed (pass) versus <15 steps completed (fail) (Humphriss et al., 2011); beanbag subtest 4–10 throws accurate (pass) or 0–3 throws accurate (fail) (poor skills defined as <1 SD from mean (Golding et al., 2014b)); threading lace 9–21 s (pass) versus 22–105 s (fail) (based on median value); peg board preferred hand 15–22 s (pass) versus 23–46 s (fail) (based on median value); peg board non-preferred hand 15–25 s (pass) versus 35–62 s (fail) (based on median value).

#### 2.2.2 Developmental coordination disorder

Children with probable DCD were identified by using DSM-IV criteria adapted for research by using the 2006 Leeds Consensus Statement as described by Lingam et al., 2009, Lingam et al., 2010. Children with probable DCD met all four DSM-IV criteria. Details of the inclusion and exclusion criteria are described in detail in Taylor et al. (2018).

### 2.3 Confounders

The mothers received four postal self-completion questionnaires during pregnancy. The questionnaires are available from the study website (http://www.bristol.ac.uk/alspac/researchers/resources-available/data-details/questionnaires/). Information collected included data on maternal education, smoking in the first trimester of pregnancy, alcohol in pregnancy in the first trimester of pregnancy, maternal age and parity.

### 2.4 Statistical analysis

For each subtest, children with an attention to task rated *poor* by the tester were excluded from the dataset. Children aged <7 or >8 years old on the test day were also excluded.

Statistical analyses were undertaken with SPSS version 23 (IBM Corp., Chicago, IL, USA). Datasets were prepared in two ways: (1) a “complete cases” dataset with inclusion of cases with complete data on exposure, outcomes and confounders (n = 1558); (2) an "all available data" dataset with inclusion of all available cases. Multiple imputation was not undertaken: few data were missing from the set of confounders and so imputation was unlikely to affect the results substantially (for example, for cases with a valid prenatal lead measurement and a complete set of outcomes the percentages of missing data on confounders were: sex 0%, maternal education attainment 3.6%, smoking 5.5%, alcohol 10.1%, age 0.6%, parity 5.4%). Data from complete cases are presented in the main paper and data from all available cases are shown in Taylor et al. (2018).

Chi-square tests were used to analyse differences in categorical data, and ANOVA was used to compare continuous values by blood lead, cadmium or mercury concentrations. Logistic regression analysis was used to examine the effect of lead below the reference value, or cadmium or mercury below the median values, on the likelihood of passing each subtest category. These models were repeated with lead, cadmium and mercury exposure as continuous variables. Logistic regression was also use to test the effects of being the lowest quartile of lead, cadmium or mercury concentration compared with the highest quartile on passing each subtest. All models were adjusted for maternal education, smoking in pregnancy, alcohol in pregnancy, age, parity, and sex of the child. The models were repeated with additional adjustment for the gestational age at which the samples were taken.

Regression diagnostics (primarily plots of residuals) were used to check that the models fitted the observed data well, to test the assumptions of regression, and to identify any cases that had undue influence on the model.

## 3 Results

### 3.1 Sample characteristics

The study flow chart is shown in Fig. 1. The characteristics of the participants included in the study according to prenatal lead, cadmium or mercury category are shown in Table 1. There were strong univariate associations for maternal education, age and smoking with all three metals, with higher educational attainment, older age and being a smoker predicting a higher blood metal category. There were no associations with parity or alcohol for cadmium; there were weak associations for parity and lead (parity = 0 was associated with blood lead ≥5.00 μg/l), and for mercury and alcohol (drinking alcohol was associated with blood mercury ≥2.00 μg/l). There was a strong association between lead and alcohol intake (drinking alcohol was associated with blood lead ≥5.00 μg/dl). There were no associations for child sex.

**Table 1:** Maternal and child characteristics: complete cases.

	Prenatal lead (μg/dl)			Prenatal cadmium (μg/l)			Prenatal mercury (μg/l)		
	<5.00 (n = 1337)	≥5.00 (n = 221)	p	<0.25 (n = 756)	≥0.25 (n = 802)	p	<2.00 (n = 770)	≥2.00 (n = 788)	p
Maternal education									
None/CSE/Vocational/O level	749 (87.8%)	104 (12.2%)	0.013	369 (43.3%)	484 (56.7%)	<0.001	498 (58.4%)	355 (41.6%)	<0.001
A level/degree	588 (83.4%)	117 (16.6%)		387 (54.9%)	318 (45.1%)		272 (38.6%)	433 (61.4%)	
Maternal age									
≤24	193 (93.2%)	14 (6.8%)	0.001	65 (31.3%)	143 (68.7%)	<0.001	143 (68.8%)	65 (31.2%)	<0.001
25–29	507 (85.8%)	84 (14.2%)		305 (51.6%)	286 (48.4%)		301 (50.9%)	290 (49.1%)	
30–34	476 (85.5%)	81 (14.5%)		291 (52.2%)	266 (47.8%)		246 (44.2%)	311 (55.8%)	
≥35	160 (79.2%)	42 (20.8%)		95 (47.0%)	107 (530%)		80 (39.6%)	122 (60.4%)	
Parity									
0	603 (83.9%)	116 (16.1%)	0.041	342 (47.6%)	377 (52.4%)	0.484	323 (44.9%)	396 (55.1%)	0.001
≥1	732 (87.5%)	104 (12.5%)		414 (49.3%)	425 (50.7%)		447 (53.3%)	392 (46.7%)	
Smoking									
No	1155 (87.2%)	169 (12.8%)	<0.001	753 (56.9%)	571 (43.1%)	<0.001	626 (47.3%)	698 (52.7%)	<0.001
Yes	182 (77.8%)	52 (22.2%)		<5%	>95%		144 (61.5%)	90 (38.5%)	
Alcohol									
No	939 (88.3%)	124 (11.7%)	<0.001	518 (48.8%)	545 (51.2%)	0.811	542 (51.0%)	521 (49.0%)	0.070
Yes	398 (80.4%)	97 (19.6%)		238 (48.1%)	257 (51.9%)		228 (46.1%)	267 (53.9%)	
Child sex									
Male	678 (85.8%)	102 (14.2%)	0.209	385 (49.4%)	395 (50.6%)	0.509	389 (50.1%)	388 (49.9%)	0.684
Female	659 (84.7%)	119 (15.3%)		371 (47.7%)	407 (52.3%)		379 (49.5%)	394 (50.5%)	

Participants with poor attention excluded.

### 3.2 Blood concentrations of lead, cadmium and mercury

For complete cases the prenatal blood concentrations were: lead 3.66 ± 1.55 (range 0.20–19.14, median 3.39) μg/dl; cadmium 0.45 ± 0.54 (range 0.14–6.30, median 0.25) μg/l; mercury 2.23 ± 1.14 (range 0.43–11.5, median 2.00) μg/l.

### 3.3 Associations with movement ABC variables

There were no univariate associations for the categories of lead, cadmium or mercury with any of the Movement ABC subtests with two exceptions: there was a weak association of cadmium with balance (heel to toe steps; children whose mothers had high cadmium levels had a higher prevalence of passing the test, p = 0.081) and of high mercury levels with manual dexterity (peg board for non-preferred hand; children whose mothers had high mercury levels had a higher prevalence of failing the test, p = 0.053) (Table 2). These two associations were maintained in unadjusted logistic regression models, but were completely attenuated on adjustment (p = 0.169 and p = 0.122, respectively; Table 3). There were no other associations shown in the adjusted models. Analyses with exposures as continuous variables showed similar results, except that the association of cadmium with balance was retained after adjustment, but there were no associations of any outcome with mercury exposure in either unadjusted or adjusted models (Table 2 in Taylor et al. (2018)). Models comparing the lowest quartile of lead, cadmium or mercury exposure with the top quartile also showed that there were no associations between the prenatal exposures and the Movement ABC subtest results in adjusted models (Table 4). Additional adjustment for the gestational age at which the prenatal blood samples were taken made very little difference to the results (Table 3 in Taylor et al. (2018)).

**Table 2:** Motor skills in children at age 7 years by category of prenatal lead, cadmium or mercury exposure: complete cases (n = 1558).

		Prenatal blood lead (μg/dl)			Prenatal blood cadmium (μg/l)			Prenatal blood mercury (μg/l)		
		<5.00	≥5.00	p	<0.25	≥0.25	p	<2.00	≥2.00	p
		n = 1337	n = 221		n = 756	n = 802		n = 770	n = 788	
Balance	Heel to toe steps									
	15 (good)	677 (85.4%)	116 (14.6%)	0.610	402 (50.7%)	391 (49.3%)	0.081	379 (47.8%)	414 (52.2%)	0.190
	< 15	660 (86.3%)	105 (13.7%)		354 (46.3%)	411 (53.7%)		389 (51.3%)	370 (48.7%)	
Ball skills	Beanbag (n)									
	4–10 (good)	1157 (861%)	187 (13.9%)	0.442	650 (48.4%)	694 (51.6%)	0.751	659 (49.0%)	685 (51.0%)	0.441
	0–3	180 (84.1%)	34 (15.9%)		106 (49.5%)	108 (50.5%)		111 (51.9%)	103 (48.4%)	
Manual dexterity	Threading lace (s)									
	9–22 (good)	722 (86.2%)	116 (13.8%)	0.676	393 (47.1%)	445 (52.9%)	0.166	401 (47.9%)	437 (52.1%)	0.181
	23–105	615 (85.4%)	105 (14.4%)		363 (50.4%)	357 (49.6%)		369 (51.2%)	351 (48.8%)	
	Peg board (s)									
	Preferred hand									
	15–22 (good)	7814 (86.5%)	122 (13.5%)	0.370	437 (48.4%)	466 (51.6%)	0.904	440 (48.7%)	463 (51.3%)	0.519
	23–46	556 (84.9%)	99 (15.1%)		319 (48.7%)	336 (51.3%)		330 (50.4%)	325 (49.6%)	
	Non-preferred hand									
	15–25 (good)	746 (86.1%)	120 (13.9%)	0.678	415 (47.9%)	451 (52.1%)	0.595	407 (47.2%)	451 (52.8%)	0.053
	26–63	591 (85.4%)	101 (14.6%)		341 (49.3%)	351 (50.7%)		361 (52.5%)	331 (47.8%)	

Manual dexterity variables categorised on median value.

Participants with poor attention to tasks excluded.

**Table 3:** Associations of motor skills with prenatal blood lead, cadmium or mercury: complete cases (n = 1558).

		Prenatal blood lead		Prenatal blood cadmium		Prenatal blood mercury	
		OR (95% CI)	p	OR (95% CI)	p	OR (95% CI)	p
Balance	Heel to toe: ref. 15 steps (vs 1–14)						
	Unadjusted	1.08 (0.81, 1.43)	0.610	0.84 (0.69, 1.02)	0.081	1.14 (0.94, 1.40)	0.190
	Adjusted	0.99 (0.74, 1.33)	0.933	0.86 (0.69, 1.07)	0.169	1.05 (0.85, 1.29)	0.663
Ball skills	Beanbag: ref. 7–10 throws (vs 1–3)						
	Unadjusted	0.86 (0.58, 1.23)	0.442	1.05 (0.79, 1.40)	0.751	1.12 (0.83, 1.50)	0.441
	Adjusted	0.88 (0.58, 1.32)	0.540	1.04 (0.76, 1.42)	0.805	1.15 (0.85, 1.56)	0.352
Manual dexterity	Threading lace: ref. 9–21 s (vs 22–105)						
	Unadjusted	1.06 (0.80, 1.41)	0.676	0.88 (0.72, 1.07)	0.195	0.87 (0.72, 1.07)	0.181
	Adjusted	1.12 (0.83, 1.50)	0.468	0.85 (0.68, 1.05)	0.129	0.89 (0.72, 1.10)	0.293
	Peg board						
	Preferred hand: ref. 15–22 s (vs 23–46)						
	Unadjusted	1.14 (0.86, 1.52)	0.371	0.99 (0.81, 1.21)	0.904	0.94 (0.77, 1.15)	0.519
	Adjusted	1.19 (0.88, 1.60)	0.256	0.95 (0.76, 1.18)	0.625	0.97 (0.78, 1.20)	0.756
	Non-preferred hand: ref. 15–25 s (vs 25–62)						
	Unadjusted	1.06 (0.80, 1.41)	0.678	0.95 (0.78, 1.16)	0.595	0.82 (0.67, 1.00)	0.053
	Adjusted	1.14 (0.85, 1.54)	0.372	0.91 (0.74, 1.13)	0.403	0.85 (0.69, 1.05)	0.122

Reference categories: lead <5.00 μg/dl, cadmium <0.25 μg/l, mercury <2.00 μg/l.

Participants with poor attention to task excluded.

Adjusted for: sex, maternal education, smoking in pregnancy, alcohol in pregnancy, maternal age and parity.

**Table 4:** Associations of motor skills with quartiles of prenatal blood lead, cadmium or mercury.

			Prenatal blood lead		Prenatal blood cadmium		Prenatal blood mercury	
			OR (95% CI)	p	OR (95% CI)	p	OR (95% CI)	p
Balance	Heel to toe: ref. 15 steps (vs 1–14)	Q4^a^	0.98 (0.73, 1.31)	0.870	0.80 (0.58, 1.11)	0.181	0.97 (0.72, 1.30)	0.823
Ball skills	Beanbag: ref. 7–10 throws (vs 1–3)	Q4^a^	1.07 (0.71, 1.63)	0.669	1.19 (0.73, 1.93)	0.486	0.86 (0.56, 1.31)	0.481
Manual dexterity	Threading lace: ref. 9–21 s (vs 22–105)	Q4^a^	1.01 (0.75, 1.35)	0.744	0.89 (0.65, 1.22)	0.478	1.24 (0.92, 1.67)	0.160
	Peg board							
	Preferred hand: ref. 15–22 s (vs 23–46)	Q4^a^	1.23 (0.92, 1.66)	0.169	0.90 (0.65, 1.25)	0.520	1.16 (0.86, 1.57)	0.333
	Non-preferred hand: ref. 15–25 (vs 25–62 s)	Q4^a^	0.99 (0.73, 1.32)	0.917	1.05 (0.76, 1.44)	0.786	0.84 (0.63, 1.13)	0.256

Reference category lead <5.00 μg/dl, cadmium <0.25 μg/l, mercury <2.00 μg/l.

Participants with poor attention excluded.

Adjusted for: sex, maternal education, smoking in pregnancy, alcohol in pregnancy, maternal age and parity.

a Reference category: Q1.

Models using all available cases (Tables 4–6 in Taylor et al. (2018)) yielded similar results, except that the associations for cadmium with balance (heel to toe subtest, p = 0.010) and for mercury with manual dexterity (peg boards, non-preferred hand, p = 0.080) were not completely attenuated after adjustment but the adjusted associations were weak (p = 0.059 and p = 0.071, respectively).

### 3.4 Associations with probable DCD

There were no univariate associations between probable DCD identified at age 7 years and prenatal lead or mercury (Table 5) except for a weak positive association of higher prenatal cadmium with an increased prevalence of probable DCD. There was no evidence for any associations of lead, cadmium or mercury with probable DCD in adjusted regression models (respectively: odds ratio 0.59 (95% CI 0.23, 1.55), p = 0.283; 1.54 (0.85, 2.80), p = 0.156; 0.92 (0.52, 1.63), p = 0.772).

**Table 5:** Associations of probable DCD scoring in children at age 7 years with prenatal blood lead, cadmium or mercury.

	Prenatal blood lead (μg/dl)			Prenatal blood cadmium (μg/l)			Prenatal blood mercury (μg/l)		
	<5.00	≥5.00	p	<0.25	≥0.25	p	<2.00	≥2.00	p
Probable DCD									
Yes^a^	49 (90.7%)	5 (9.3%)	0.307	20 (37.0%)	34 (63.0%)	0.085	29 (53.7%)	25 (46.3%)	0.552
No	1241 (85.8%)	205 (14.2%)		708 (49.0%)	738 (51.0%)		717 (49.7%)	729 (50.4%)	

a Probable DCD: <15th centile for Co-ordination test at age 7 years + failed Key Stage 1 writing and/or <15th centile of 23-item ADL scale (excluded IQ < 70, visual impairment, neurological condition) (Lingam et al., 2010).

## 4 Discussion

We found no evidence for an adverse association of prenatal lead, cadmium or mercury exposure with motor skills measured at age 7 years. Further, there were no associations with the functional outcome of probable DCD, identification of which is based on motor skills impacting on activities of daily living and/or educational attainment.

The role of exposure to lead, cadmium and mercury, including prenatal exposure, on cognitive development has been relatively well studied. In contrast, motor skill development has been relatively neglected, even though impairment can affect important functions (ADL) and if severe can lead to DCD, which is itself associated with impairments in other areas of development, such as mental health and educational attainment. Primary prevention by minimising exposure to heavy metals during pregnancy and childhood is preferable, but even early detection and diagnosis can enable timely and appropriate interventions and therapies. Other factors that have been implicated in motor skill development include deficiencies of essential trace metals, exposure to other pollutants, social drugs, dietary factors, maternal disorders and medications, obstetric and neonatal outcomes, social circumstances and general child health (Golding et al., 2014a).

Overall, the few studies of the associations of prenatal exposures to lead, cadmium and mercury with children's motor skills are insufficient, and too heterogeneous, to draw firm conclusions (Golding et al., 2014a). The majority of the studies have focussed on mercury and the results have generally indicated adverse associations, in contrast to our findings. For example, a study in Croatia with 135 children with motor skill assessment with the Bayley Scales of Infant Development (BSID)-III Psychomotor Development Index (PDI) at 18 months showed that cord blood mercury levels similar to the prenatal levels in the present study (median 2.98 μg/l) were associated with poorer fine motor skills (Prpic et al., 2017); in Poland, a study of 374 children also using the BSID-III PDI at 12, 24 and 36 months old showed a weak association with cord blood mercury levels (≤0.90 versus >0.90 μg/l) at 12 months, but not at 24 or 36 months, indicating that there may be a some temporality in the association (Jedrychowski et al., 2007). Studies from the Faroe Islands, however, found adverse effects of high cord blood mercury levels (geometric mean 22.5 μg/l) in children up to the age of 14 years using suites of tests including simple reaction time, tapping speed, and hand-eye co-ordination tests (Debes et al., 2006; Grandjean et al., 1997). In contrast, studies from the Seychelles Child Development Study, where mercury exposure comes from fish rather than whale-meat consumption as in the Faroes, but again with a suite of measures of motor skills (BSID, grooved pegboard, finger tapping test, trail-making etc.), and up to 19 years old, have consistently shown no associations with maternal hair mercury level at birth (median 5.9 μg/g) (Davidson et al., 1995; Davidson et al., 2000; van Wijngaarden et al., 2013).

There are even fewer studies on associations with prenatal lead or cadmium exposure. In a study in Korea in which prenatal exposure to lead and cadmium was measured at two points in pregnancy, enabling exploration of the effect of timing of exposure and interaction effects, 884 infants were assessed with the psychomotor scores of the BSID at age 6 months: there were no associations with early gestation blood cadmium or lead levels (geometric mean 1.36 μg/dl and 1.52 μg/l, respectively), nor with late gestation lead levels (1.27 μg/dl). There was a weak association, however, of late gestation cadmium level (1.52 μg/l) with motor skills and evidence for a synergistic effect modification between lead and cadmium in late pregnancy (Kim et al., 2013). Evidence from the Port Pirie study in Australia where prenatal lead exposure was high (9.5 μg/dl) supports an association with deficits in visual-motor development at age 7 years (measured with the Beery Developmental Test of Visual-Motor Integration) (Baghurst et al., 1995).

Some of the ALSPAC Co-ordination subtests have been used in previous studies of prenatal exposure to heavy metals. Using the ball skills subtest of the Movement ABC, Golding et al. (2014b) found no association with prenatal exposure to either lead, cadmium or mercury using an “exposome” approach in which multiple predictors were examined. The heel to toe balance subtest was used as part of a suite of measures of balance at age 7 and 10 years in association with prenatal cadmium and lead exposure, in which no associations were found (Taylor et al., 2015). We have extended these studies to look in further detail at the associations of the exposures with all of the five subtests of the Movement ABC score, as well as with a functional indicator, probable DCD, and also found no associations.

There are several aspects of study design that may account for the conflicting results found in these different studies. These include: (1) the measure chosen to reflect prenatal exposure (e.g. maternal blood, cord blood, placental tissue, maternal hair, etc.); (2) the timing of the measurement during gestation, which may have differing impacts on fetal neurodevelopment; (3) the magnitude of exposure; (4) the tests used for measurement of motor skills, which may measure differing abilities within the category of motor skills and may have different sensitivities and validities (e.g. BSID-II or -III PDI, Beery Developmental Test of Visual-Motor Integration, Finger Tapping Test, Motor Skills Test in the Gesell Development Schedules, McCarthy Scales of Children's Abilities, etc.); (5) the age of the child at testing and the number of children included; (6) differences in the settings of the studies (the environmental source of the exposure and co-exposures may be important); (7) variations in data handling and statistical analyses.

There are several strengths of this study. (1) The study involved large numbers of pregnant women with measures of prenatal lead, cadmium and mercury, and child motor skills measured at 7 years. (2) The prenatal exposure was measured in the first half of pregnancy, in contrast to studies that have relied on cord blood levels or other matrices such as hair or urine. (3) The motor skills measure used in the study is well validated and was conducted by trained examiners with supervision. The Movement ABC test is frequently used in clinics by health professionals to assess children with symptoms of delayed or impaired development and is a recognised standardised assessment tool used worldwide. (4) Even under the supervision of trained examiners, tests are subject to error, based for example, on the child's boredom, mood, tiredness, and rapport with the examiner. However, to address this we were able to exclude children whom the examiner judged had a poor attempt at the task, although the vast majority of children were rated good.

There are also several limitations. (1) The Movement ABC test has been used less frequently as a research tool to measure motor skills than the BSID, which limits comparisons with other studies. However, the BSID is only suitable for children aged up to 42 months, so would not be appropriate for our study population. In addition, we were unable to conduct all of the subtests due to time constraints, which meant that we were unable to adhere strictly to the protocol for calculating official Movement ABC scores. The test is unable to distinguish between fine and gross motor skills as the BSID-III PDI does. (2) Children with queried trials (peg game and threading lace) might have more motor difficulties than children without queried or failed trials, causing bias. (3) Although we were able to account for many possible confounders in our analyses, there are likely to be others that were unable to adjust for. This would contribute to any findings being due to chance. (4) The numbers of cases identified as having DCD were small and limit the conclusions that can be drawn from this part of the investigation. (5) The role of concomitant exposure to other pollutants and other factors that may be associated with motor skills might have the effect of masking associations was not included and should be explored further in other studies. (6) The time lapse between the exposure and the outcomes in this study means that the child will have experienced unknown levels of further exposure to lead, cadmium or mercury during childhood, which was not accounted for.

## 5 Conclusion

We did not find any evidence to support associations between moderately low prenatal exposures to lead, cadmium or mercury and motor skills at age 7 years. Motor skills have been relatively neglected compared with cognitive outcomes in association with exposure to heavy metals, although they can have a profound impact on children's lives. Further study of the role of exposure to heavy metals both prenatally and postnatally in motor skill development in large trials with well-defined measures of exposure, and validated and standardised tools to measure outcome, is essential. Early identification of symptoms of motor skill impairment is also important to enable prompt investigation and treatment.
